# Strengthening Effect of Nb on Ferrite Grain Boundary in X70 Pipeline Steel

**DOI:** 10.3390/ma14010061

**Published:** 2020-12-25

**Authors:** Zhongyi Li, Zhipeng Li, Wenhuai Tian

**Affiliations:** School of Materials Science and Engineering, University of Science and Technology Beijing, Beijing 100083, China; lizhongyii@sina.com (Z.L.); zplmse@ustb.edu.cn (Z.L.)

**Keywords:** grain boundary, 3D atom probe, electron energy loss spectrum, first principles, valence electron structure

## Abstract

Understanding the strengthening effect of niobium on ferrite grain boundaries from the perspective of valence electron structures will help to use niobium and other microalloying elements more effectively to improve the performance of steel materials. In this paper, the effect of niobium element on ferrite grain boundary strengthening is studied based on microstructure analysis at the nanometer scale. The enrichment of niobium in pipeline steel at ferrite boundary was observed by a three-dimensional atomic probe test. Segregation of Nb is observed in the ferrite grain boundaries of X70 steel, and its maximum concentration is 0.294–0.466 at.%. The charges in the occupancy of the Fe 3d state in grain and grain boundary were 7.23 and 7.37, respectively, based on quantitative analysis of electron energy loss spectra (EELS). The first-principle calculation suggests that the charges in the occupancy of 3d state for grain boundary iron are 6.57 and 6.68, respectively, before and after the Nb doping (with an increase of 1.67%), which reveals a similar trend to that of the EELS results. Through Nb alloying, the 3d valence electronic density of the state of Fe in grain boundary moves to a lower energy, which can reduce the total energy of the system and make the grain boundary more stable. Meanwhile, the charges in the occupancy of the 3d state for Fe in the grain boundary increases, providing more electrons for grain boundary bonding. These improve the strength and toughness of the material. This work provides a fundamental understanding for pipeline steel strengthening by element alloying.

## 1. Introduction

Natural gas is one of the most important sources of clean energy at present. From 2007 to 2018, the annual compound growth rate of natural gas consumption in China reached 12.9% [1]. The scale of China’s oil and gas pipeline network will reach 169,000 km in 2020 and 240,000 km in 2025. Increasing the diameter of the pipeline and the transmission pressure is the development trend for long-distance and mass transport of gas, which requires higher strength and toughness pipeline steel materials [2]. In order to improve performance, different alloying elements, such as molybdenum, nickel, copper, vanadium, niobium, etc., are added to the pipeline steel [3,4]. After many experiments, researchers gradually reduced or eliminated the use of alloying elements such as molybdenum and nickel. The simpler chemical composition design based on C-Mn-Nb realized the industrial mass production of X70 steel [5]. On the one hand, it reduces the complexity of the component design and reduces the cost; on the other hand, it maintains high strength and toughness. The average tensile strength of X70 steel with a thickness of 17.5 mm is 93 MPa and 38 MPa higher than the lower limits of 570 MPa and 625 MPa required for the tensile strengths of X70 and X80 steel in the API SPEC 5L standard, respectively. The ductile brittle transition temperature is lower than −60 °C. The improvement of steel performance is determined by many factors such as chemical composition, microstructure, and rolling and cooling process. Niobium microalloying is one of the most characteristic factors in composition design to improve the properties of this industrial X70 steel. During hot rolling, the precipitation of niobium carbonitride can pin the grain boundaries [6,7] and solid solution niobium can inhibit the migration of austenite grain boundaries through solute drag [8,9,10]. Both methods can delay recrystallization and refine grains to improve performance [11]. There have been many studies on the precipitation of niobium; however, for solid solution niobium, due to the difficulty of the detection test, there are few studies on it. The mechanism of niobium strengthening in pipeline steel at the atomic scale is also unclear, which may hinder the use of niobium for further optimization of steel’s mechanical properties.

Shrestha et al. used in situ heating transmission electron microscopy to study the resistance of niobium to the movement of dislocations during 575 °C aging [12]. Li et al. used Auger electron spectroscopy to detect the segregation of niobium at the original austenite grain boundary of the forging sample [13]. The competition mechanism proposed by Messmer et al. can explain grain boundary strengthening [14]: electronegative impure atoms acquire electrons from surrounding metal atoms and weaken grain boundary bonding, while electropositive impure atoms transfer electrons to adjacent metal atoms to enhance grain boundary bonding. Other researchers [15,16,17] used first-principles and discrete variational methods to study the effects of niobium, molybdenum, and chromium atoms on the grain boundary bonding force of iron.

Sharp peaks known as “white lines” are observed at the L_2_ and L_3_ absorption edges in the electron-energy-loss spectra (EELS) of transition metals. These white lines originate from excitations of 2p core electrons in an atom to unoccupied d states near the Fermi level. Since the shape of the electron energy loss spectrum is related to bonding and valence of atoms, it is used to analyze that in the electronic structure of elements. Muller et al. found that, in the electron energy loss spectrum, the electron structure and the fracture properties of the material are related when studying NiAl alloys. When the 3d valence electron state density of atoms at the grain boundary is lower than that in the grain, the bonding strength of the grain boundary is higher than that in the grain and the grain boundary exhibits toughness [18]. Pearson et al. used the L2 and L3 peaks in the electron energy loss spectrum to measure the valence electron structure of CuZr alloys [19]. Although the research objects are different, from the results of the two researchers, there is an inherent connection between the valence electron structure and the performance of the material. Therefore, it is speculated that the 3d valence electron density of state for iron at grain boundaries in pipeline steel can be used to judge the strength of grain boundary bonding. The charges in the occupancy of 3d state for iron can be obtained by the method of first-principle calculation and electron energy loss spectrum test. It is feasible to analyze the micro-mechanism of the strengthening effect of niobium on the ferrite grain boundary from the transfer of change, the increase or decrease of the charges in the occupancy of 3d state, and the change in distribution of 3d electronic density of states. When this quantitative analysis method related to electronic structure is applied to optimization of the material chemical composition design, it is possible to qualitatively evaluate and compare the effect of elements on grain boundary strengthening. Reaz et al. reported that changes in magnetism of Fe-based materials due to interface/grain-boundary properties change but without atomistic calculation to justify the origin [20,21]. The first-principles analysis method can be used to answer this question and has more applications in similar studies.

In order to understand the strengthening effect of Nb on ferrite grain boundary at the atomic scale, this work firstly confirms whether there is grain boundary segregation of niobium in X70 pipeline steel through a three-dimensional atom probe test; secondly, in order to understand the influence of niobium on neighboring iron, the electron energy loss spectra (EELS) of the grain boundary and intragranular iron was measured. Then, we calculated the charges in the occupancy of the 3d state for iron at the grain boundary and compared it with that in the grain. It is revealed that charges in the occupancy of the 3d state for atoms at the grain boundary are higher than that inside the grain, indicating that grain boundary bonding has been enhanced and that the grain boundary shows toughness [18,19]. Finally, first-principles calculation is used to analyze the distribution of the 3d valence electron density of state, the charges in the occupancy of the 3d state, and the charge distribution changes caused by alloying.

## 2. Materials and Methods

### 2.1. Experimental Materials

The test material is taken from an industrially produced X70 pipeline steel plate coil, and the thickness of the steel plate is 17.5 mm. The chemical composition of the material is (wt.%) 0.065 C, 0.20 Si, 1.55 Mn, 0.065 Nb, 0.016 P, and 0.0012 S. The average tensile strength of the steel is 663 MPa, the average yield strength of the steel is 536 MPa, and the Charpy V impact energy at −60 °C is greater than 380 J [5]. Young’s modulus of the material is 206 GPa, and Poisson’s ratio is 0.28.

### 2.2. Microstructure Analysis

A thin film with a thickness of about 0.60 mm was fabricated from the steel sheet. Thin foil specimens for transmission electron microscopy (TEM, FEI, Hillsboro, OR, USA) and electron energy loss spectroscopy (EELS, JEOL, Tokyo, Japan) analysis were mechanically polished to about 50 μm thickness, punched to disks of 3 mm diameter, and then electropolished in the solution that contains 10% perchloric acid and 90% glacial acetic acid. At a temperature of −30 °C and a current of 35–160 mA, electrolytic double spray was used for 2 to 3 min. TEM observation was performed in a FEI Tecnai G2 F20 (Hillsboro, OR, USA) field emission transmission electron microscope operated at an accelerating voltage of 200 kV.

The 10% perchloric acid alcohol solution was used for electrolytic polishing of scanning electron microscope (SEM, JEOL, Tokyo, Japan) samples, with voltage of 26 V, current of 0.6 A, and polishing time of 25–30 s. The boundary properties were examined by electron back-scattered diffraction (EBSD) using a JEOL JEM-7800F field emission scanning electron microscope equipped (Tokyo, Japan) with an Oxford BESD detector (Oxford, UK). The EBSD data were analyzed using CHANNL 5 software (HKL, Danbury, CT, USA).

### 2.3. Three-Dimensional Atom Probe Test

In order to analyze whether niobium is enriched at the X70 ferrite grain boundary, the three-dimensional atom probe system CAMECA LEAP4000HR 3DAP (three-dimensional atom probe, Madison, WI, USA) was used to test the pipeline steel samples. Since the focused ion beam may affect the segregation of solute atoms to dislocations, electrolytic polishing was used to prepare the tip. Firstly, a small rod with a size of 0.3 mm × 0.3 mm × 10 mm was cut from the steel sheet and was electrolytically polished with 25% perchloric acid in acetic acid solution. Then, 2% perchloric acid was used in ethylene glycol butyl ether solution for secondary electropolishing. The temperature of the sample during 3DAP analysis was set to 40–60 K, the pulse fraction of the ionization evaporation voltage was 20%, and the pulse frequency was 200 KHz. Ivas 3.6.12 software was used to reconstruct and quantitatively analyze the experimental data.

### 2.4. Electron Energy Loss Spectrum Measurement

The enrichment of niobium at the grain boundary could affect the 3d valence electron state density distribution of iron at the grain boundary. The change in the charges in the occupancy of the Fe 3d state, which indicates the degree of influence, can be obtained by detecting the fine structure of the EELS of iron atoms in the grain boundaries and grains. The samples were prepared by electrochemical polishing, and a JEOL ARM-200F (Tokyo, Japan) cold field emission spherical aberration correction electron microscope was used to collect the electron energy loss spectra (EELS) of the grain boundaries and in-grain iron of X70 steel samples.

The exponential fitting method was used to subtract the background of the collected electron energy loss spectrum, and the Fourier ratio method was used for deconvolution to eliminate the influence of the sample on the multiple scattering of electrons. Finally, the white line of iron in the electron energy loss spectrum was processed [22] to obtain the charges in the occupancy of the 3d state for Fe.

### 2.5. First-Principles Calculation

**Figure 1:**
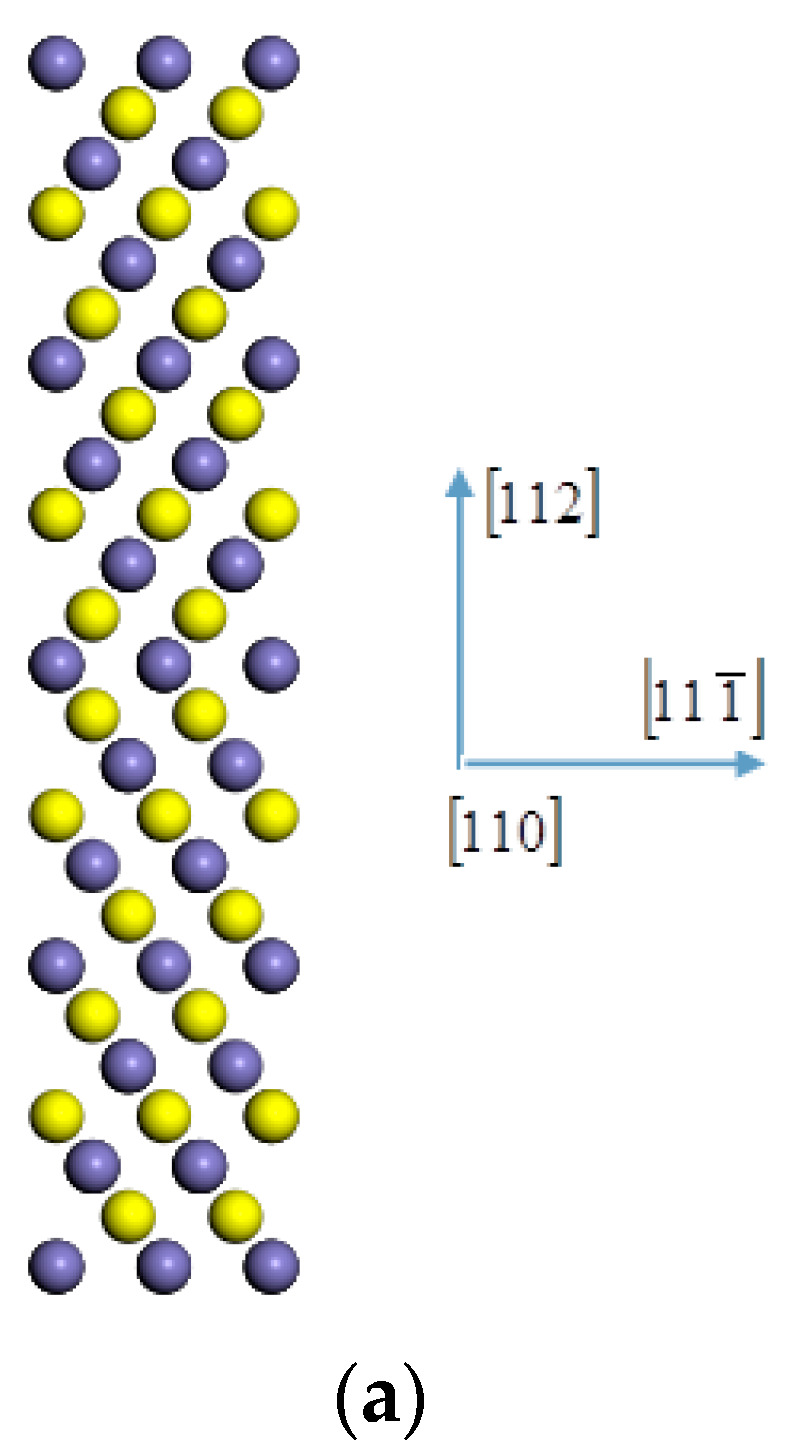

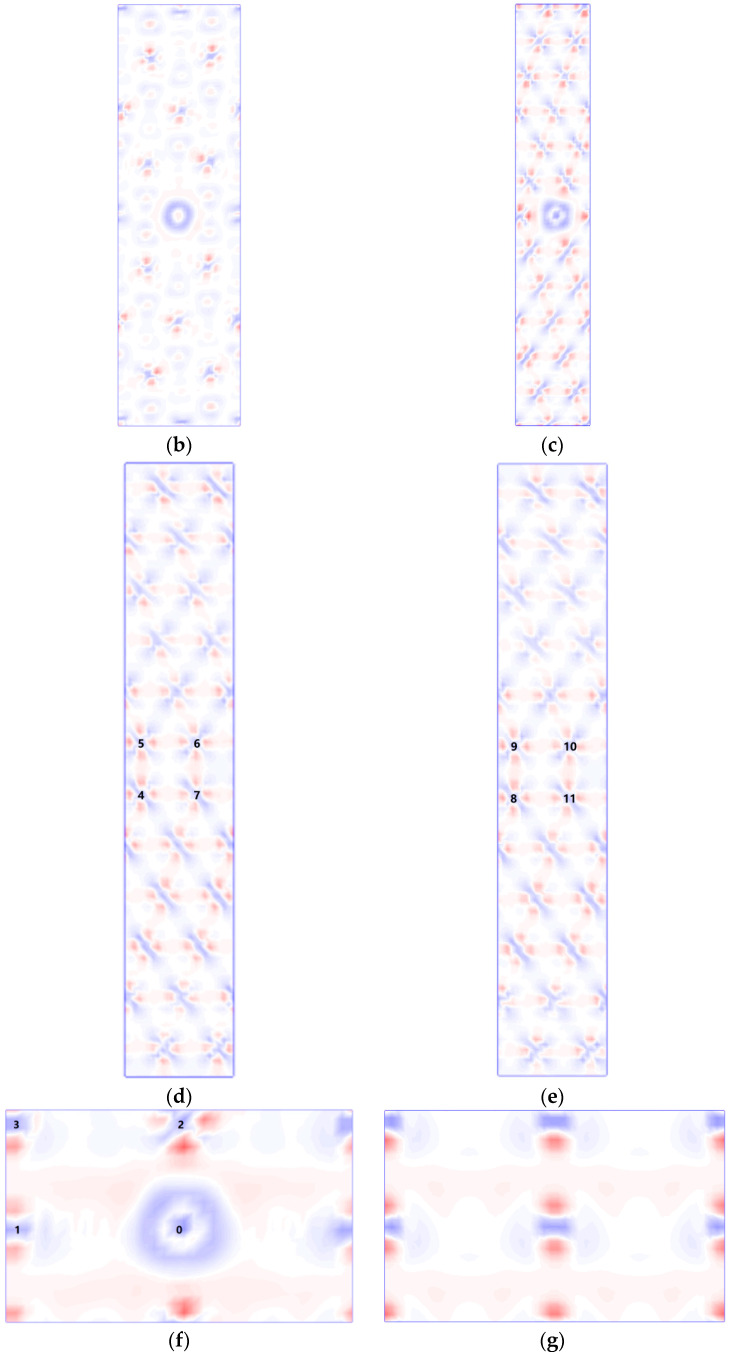
(**a**) The atomic configurations of bcc Fe for Σ3[110] (112) grain boundary, the charge density difference on the Nb-doped (**b**) (110) plane, (**c**) (111) plane, (**d**) (111) plane, (**e**) (111) plane, and (**f**) (112) plane and Nb-undoped (**g**) (112) plane of αFe Σ3[110] (112) grain boundary: red indicates the inflow of electrons; blue indicates the outflow of electrons.

The first-principles spin-polarized calculations were carried out using density functional theory as implemented in the CASTEP software (Accelrys, Cambridge, UK) to obtain most of the information including charges in the occupancy of the 3d state of iron [23,24]. Firstly, an initial ferrite symmetric tilting grain boundary model was built. The grain boundary model is far less complex than the actual grain boundary. In order to reduce this gap, we first analyzed the grain boundary characteristics of X70 steel and included the grain boundary characteristics in the model. The low-index coincidence site lattice grain boundary that accounts for the largest proportion of large-angle grain boundaries of X70 pipeline steel is the Σ3 coincidence site lattice according to the results of the SEM test. Compared with the Σ3(111) grain boundary of bcc iron, the energy and structure of Σ3(112) grain boundary of bcc iron indicate that it is a stable grain boundary which can be used for further research [25,26]. The [110] (112) symmetric tilting grain boundary model was established [27], as shown in Figure 1a; the atoms at different adjacent layers along the [110] direction were marked with different colors, the [110] crystal orientation is the axis of rotation, the (112) plane is the grain boundary, and the angle between the crystals on both sides is 109.47°, which is equivalent to a rotation of 70.53° due to secondary axis symmetry. The supercell used in the model calculation contains 96 atoms, and the size is 8.10 × 4.96 × 28.08 Å^3^. In the model, the lattice constant of ferromagnetic α-iron is 2.86 Å, which is in good agreement with the experimental value of 2.867 Å [28]. The initially constructed grain boundary model was relaxed to optimize the position of all atoms. The iron atom in the grain boundary (Figure 1g) was replaced with niobium atom, as shown in Figure 1f at the position marked “0”. Figure 1b,c show the (110) and (111) planes containing niobium atom, which are perpendicular to the grain boundary. For geometry optimization, the shape and volume of the supercell were fixed while the atomic positions were allowed to relax until the forces on each atom were less than 0.05 eV/Å. For the exchange-correlation function, the generalized gradient approximation (GGA, PBE) was used. The self-consistent field loop converges to $1\times 10^{-6}\;\mathrm{eV}/\mathrm{atom}$. The total energy of the self-consistent calculation converges to $1\times 10^{-5}\;\mathrm{eV}/\mathrm{atom}$. The ultra-soft pseudopotential was adopted with the maximum cutoff energy of 300 eV and K grid of 6 × 6 × 1.

## 3. Results and Discussion

### 3.1. Grain Boundary Characteristics

It is generally believed that both large-angle grain and small-angle grain boundaries could contribute to strengthening of materials; moreover, the large-angle grain boundaries, especially some special large-angle grain boundaries, such as low-index coincident site lattice (CSL) grain boundaries, can effectively restrain dislocation movement and crack propagation, thereby improving the strength and toughness of steel [29,30]. The purpose of this experiment was to obtain the proportion of large-angle grain boundaries and the low-index coincident site lattice grain boundaries.

**Figure 2:**
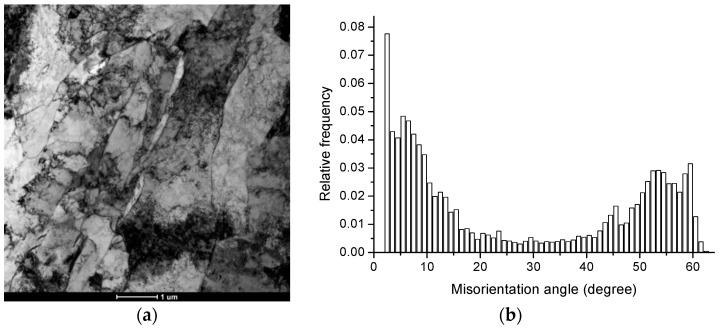
(**a**) The bright field TEM image shows the acicular ferrite of X70 steel, and (**b**) The distribution of misorientation angles showing a “bimodal” distribution in two areas of 2°~15° and 45°~60°.

The microstructure of X70 steel is acicular ferrite, as shown in Figure 2a.

**Figure 3:**
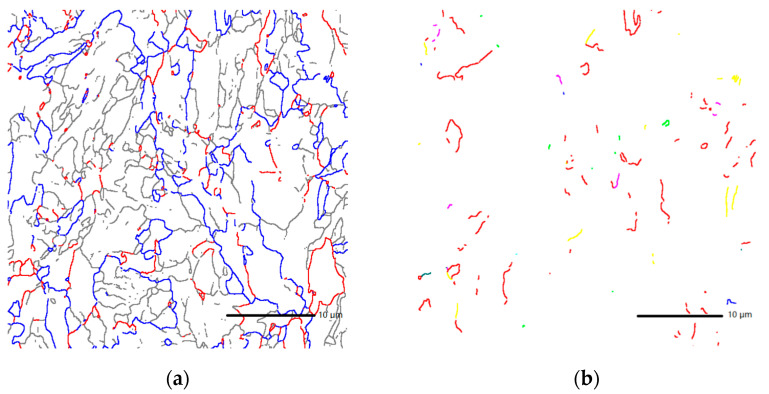
(**a**) The grain boundaries of 0–15°, 15°–45°, and greater than 45° are shown in the gray, red, and blue lines, and (**b**) Σ3, Σ11 CSL of the X70 steel are shown in the red and yellow lines.

The results of scanning electron microscopy experiments show that there is a preferential distribution of grain orientation differences in steel. There is a “bimodal” distribution in two areas at 2°–15° and 45°–60°, as shown in Figure 2b. The grain boundaries of 0–15°, 15°–45°, and greater than 45° account for 49.8%, 16.7%, and 33.5%, respectively, as shown in the gray, red, and blue lines in Figure 3a.

The test results also show that there are ∑3, ∑11, and other low-index coincident site lattice grain boundaries in steel, as shown in the red and yellow lines in Figure 3b; most of them are ∑3 coincident site lattice grain boundaries. The proportions of ∑3 coincident site lattices in the large-angle grain boundaries above 15° and 45° are 14.2% and 21.3%, respectively. Four samples were examined by electron back-scattered diffraction (EBSD). The EBSD results of all samples were consistent in the “bimodal” distribution and in the proportions of ∑3 coincident site lattices in the large-angle grain boundaries.

### 3.2. Elements Distribution Near Grain Boundaries

**Figure 4:**
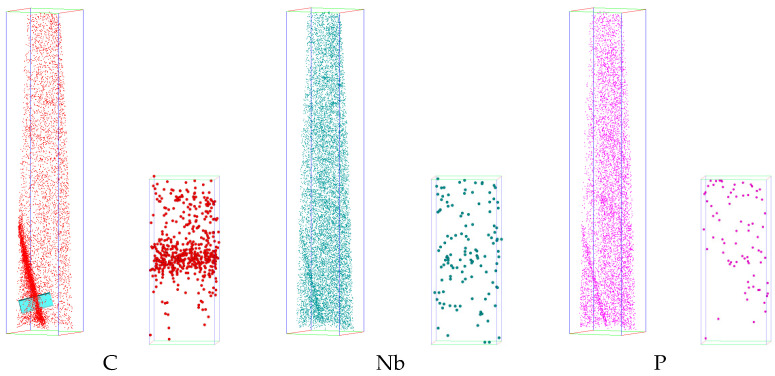
Three-dimensional elemental maps of the box (54 nm × 54 nm × 298 nm) containing grain boundary and magnified maps of enriched atoms across the boundary.

The distribution of niobium at the grain boundary was observed in X70 steel by the three-dimensional atom probe test, as shown in Figure 4.

Enrichment of niobium, carbon, and phosphorus can be observed at the grain boundary. The one-dimensional concentration distribution diagram of carbon, niobium, and phosphorus across the analyzed grain boundaries (the inserted box in Figure 4) is shown in a, which shows that the degree of carbon segregation is the largest, followed by niobium and phosphorus.

**Figure 5:**
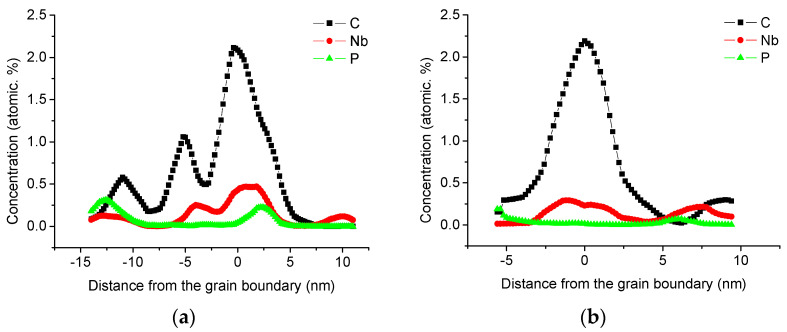
Concentration profiles along the direction perpendicular to the boundary, (**a**) sample 1, (**b**) sample 2.

The one-dimensional element concentration distribution diagram of the grain boundary of the second sample is shown in Figure 5b. The two tip samples were from different parts of the same steel sheet. The maximum atomic concentration of niobium at the grain boundary of this sample is 0.294%, which is approximately the niobium atom at the grain boundary detected by the previous sample at 63% of the maximum concentration.

Table 1 shows the chemical composition analysis results of X70 steel, the grains, and the local chemical composition results of the grain boundaries measured by the three-dimensional atom probe.

The atomic percentage content of carbon, niobium, and phosphorus at the grain boundary is much higher than that in the grain. The maximum atomic percentage content of niobium in the grain boundary reaches 0.294 and 0.466 at.%, which is 7.5–11.9 times the niobium content of 0.039 at.% in the matrix, and the enrichment area is several nanometers; carbon and phosphorus also have obvious segregation at the grain boundaries. When carbon atoms are dissolved in the grain boundary, the theoretical fracture strength of the grain boundary increases with the increase in carbon concentration, but the attraction of C atoms to Σ3(112) grain boundaries is less attractive than to dislocations [31]. Pipeline steels usually experience the piping process followed by the coating process at 250 °C, and 3DAP results revealed the segregation of carbon atoms along dislocation lines in the ferrite region of both UOE (bending by U press, forming by O press, Expansion) pipes and coated pipes. It is believed that segregation of carbon along dislocations in pipeline steel usually causes strain aging phenomena, such as discontinuous yielding, decrease in yield strength, and uniform elongation after the pipe-making and coating processes [4,32]. Phosphorus exists in the form of solid solution when the segregation concentration of the grain boundary is low. When the segregation concentration is high, it forms a structure similar to Fe3P, which causes a significant increase in the brittleness of the grain boundary and reduces the toughness of the material [33]. Therefore, in order to improve the strength and toughness of the material, it is necessary to reduce the segregation of carbon and phosphorus in X70 pipeline steel. When niobium is at the grain boundary, it changes the local chemical composition and structure, which will strengthen or weaken the bond of the grain boundary, thereby affecting the strength and toughness of the material.

### 3.3. Electron Energy Loss Spectrum and Charges in the Occupancy of Fe 3d State

**Figure 6:**
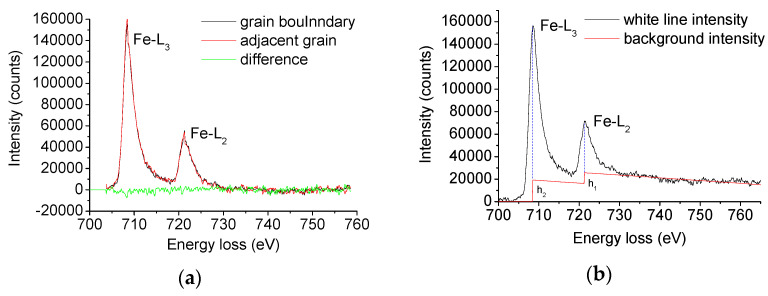
(**a**) Electron energy loss spectra (EELS) profiles of grain and grain boundary in X70 steel and (**b**) double step continuum mode.

EELS results were quantitatively analyzed to study the occupancy of the Fe 3d state, which is closely related to the bonding strength of grain boundaries according to the method given by Pearson et al. [22]. The electron energy loss spectrum of the grain boundary and intra-grain iron in X70 steel is shown in Figure 6a.

The spectrum was deducted and deconvoluted. The spectrum further reduced the background using the double step function, as shown in Figure 6b.

Table 2 shows the charges in the occupancy of the 3d state in the grain boundary and adjacent grains on both sides calculated from the energy loss spectrum.

Three sets of data are listed in the table. Each set includes the charges in the occupancy of the 3d state for Fe calculated from the electron energy loss spectra collected from the grain boundary and two adjacent crystal grains. The average charges in the occupancy of 3d state for Fe in the grain boundary and adjacent grains of X70 steel are 7.37 and 7.23, respectively. The charges in the occupancy of the 3d state for iron by the grain boundary are about 1.94 ± 0.80% higher than the grains on both sides.

### 3.4. Segregation Energy

The grain boundary energy, $\gamma_{gb}$, is defined as the difference between the potential energy $E_{gb}$ of *n* atoms in the supercell containing grain boundaries and the potential energy $E_{p}^{o}$ of a computational cell with the same number of atoms in a perfect crystal, divided by the cross-sectional area, S, of the grain boundary plane (Equation (1)):

(1) $$\gamma_{gb}=\;\left(E_{gb}-E_{p}^{o}\right)/2S$$

The calculated grain boundary energies of ∑3(112) of bcc iron are 0.57 $J/m^{2}\;$. The calculated result of the interface energy is similar to those of other authors [26]. According to the Rice and Wang models, material fracture is the result of competition through dislocation emission and brittle interface fracture [34]. The influence of solute atoms on intergranular fracture can be determined by calculating the formation energy of impure atoms on the grain boundary and surface.

The grain boundary formation energy is the difference between the total energy of the grain boundary system and the free surface energy that forms the grain boundary [27] (Equation (2)):

(2) $$E_{form}^{clean}=\left(E_{GB}^{nFe}-2E_{relaxed\;FS}^{\frac{n}{2}Fe}\right)/2A$$

$E_{GB}^{nFe}$ is defined as the difference between the total energy of the crystal and the sum of the energy of all the individual atoms composing the crystal in a free state. $2E_{relaxed\;FS}^{\frac{n}{2}Fe}$ is the energy of the two free surfaces forming the grain boundary separated from each other and relaxation. A is the cross-sectional area of the grain boundary. The factor of 2 originates from the fact that one supercell contains two grain boundaries due to periodic boundary conditions. When niobium is doped at the grain boundary, we get Equation (3):

(3) $$E_{form}^{GB}=\left(E_{GB}^{nFe+Nb}-E_{relaxed\;FS}^{\frac{n}{2}Fe+Nb}-E_{relaxed\;FS}^{\frac{n}{2}Fe}\right)/2A$$

The difference between the formation energy of grain boundaries with and without impurities is defined as the segregation energy of impure atoms at the grain boundaries, $\Delta E_{SE}^{GB}$. “Positive/negative” can be used to characterize the “weakening/strengthening” effect on the grain boundaries caused by impure atoms [15,17] (Equation (4)).

(4) $$\Delta E_{SE}^{GB}=\;E_{form}^{GB}-E_{form}^{clean}$$

$\Delta E_{SE}^{GB}$, $\Delta E_{SE}^{bulk}$, and $\Delta E_{SE}^{FS}$ are the segregation energies when the impurities are located at the grain boundary and at the positions within the grain and surface, respectively. Niobium atoms replace the positions marked “0” in the grain boundary (Figure 1f), the positions in the grain of the sixth atomic layer from the grain boundary (Figure 1a), and the surface model. The calculations of $\Delta E_{SE}^{bulk}$ and $\Delta E_{SE}^{FS}$ are similar to Equation (4). After structural optimization, the calculated segregation energy and impurity formation energies at the grain boundary, in the grain, and on the surface are shown in Table 3. For comparison between different positions of the same model, we converted the unit of $E_{form}$ and $\Delta E_{SE}$ to eV, where the denominator 2A was replaced by 2.

From the calculation results in Table 3, it can be seen that the impurity formation energy of the system is the lowest when niobium atoms are at the grain boundary compared with the surface and the crystal grains. From an energy point of view, niobium atoms tend to segregate in the grain boundaries of ferrite. When the niobium atom is at the grain boundary, the segregation energy of the system is −7.12eV, which reduces the grain boundary energy and enhances the grain boundary cohesion.

### 3.5. Charge Distribution

Elements that reduce toughness gain electrons from adjacent metal atoms and weaken the bond with adjacent atoms [14]. On the contrary, elements with increased toughness transfer electrons to adjacent metal atoms to strengthen the bond with adjacent atoms. The differential charge density diagram intuitively describes the bond formation and charge gains and losses between atoms.

The differential charge density is defined as the difference between the charge density of the atoms in the doping system and the charge density of the corresponding free atoms. The calculation results show that the differential charge density between niobium atoms and the nearest neighbor iron atoms on the (112) crystal plane (Figure 1f) and the (111) crystal plane on both sides of the grain boundary (Figure 1d) is the strongest.

The electron is transferred from the niobium atom to the nearest neighbor iron atom. When the (111) plane of the nearest neighbor (Figure 1d) and the second nearest neighbor (Figure 1e) to the Nb atom, on both sides of the grain boundary, and the common valence electrons of iron atoms increase, such as the 4–5, 7–6, 8–9, and 11–10 bonds, the iron atom bond across the grain boundary is strengthened, producing the effect of strengthening the grain boundary.

Table 4 shows the change of bond length between adjacent iron atoms of niobium before and after doping.

The bond length of the 0–1 bond on the (112) crystal plane at the grain boundary is reduced by 0.85%. On the (111) crystal plane, the bond lengths of the 4–5, 7–6, 8–9, and 11–10 bonds across the grain boundary are reduced by 0.99% to 1.45% and the 6–10, 7–11, 5–9, and 4–8 bonds that do not cross the grain boundary on the crystal plane (112) are reduced by 0.56% to 1.09%. The iron atoms that are close to niobium atoms, including those that cross grain boundaries and those that do not, have reduced bond lengths and are closer to each other.

Mulliken population means that the electric charge is distributed among the constituent atoms, which can determine the strength of the chemical bond between different atoms. Table 5 shows Mulliken electronic populations of Nb and its neighbor Fe atoms on the (110) crystal plane.

Other researchers calculated the valence electron structure of metallic iron to be 3d^6.267^4s^0.790^4p^0.943^ [35], which is close to the charge distribution on the iron atom orbital when it is undoped in Table 5. The charge distribution results show that the Nb atom loses 1.25 valence electrons; the iron atoms at positions 1 and 4 to 7 closest to the Nb atom get 0.08 to 0.16 electrons. The electron is transferred from the niobium atom to the nearest neighbor iron atom. For the iron atom closest to niobium, the electrons in the s and d orbitals increase while the electrons in the *p* orbital decrease. The *p*-orbital has strong directivity, and the decrease of electrons in the *p*-orbital are beneficial to improving the toughness of the material [36]. The charges in the occupancy of the 3d state for the closest neighbor iron at the grain boundary increased by 1.67% from 6.57 to 6.68. The absolute value and increase were close to the measured values of the electron energy loss spectrum of 7.23, 7.37, and 1.94%, respectively. Compared with Mulliken charge, Bader charge is based on a more reasonable definition of interatom boundary, the charge enclosed within the Bader volume is a better approximation of the total electronic charge of an atom.

### 3.6. Electronic Density of State

Before adding niobium, the iron atom at position 0 in Figure 1f and the iron atom at the adjacent position have the same electronic density of states, and the lowest energy of the electronic density of state is higher than −10 eV. After niobium is added, in the range of −53.5–−55.7 eV, −29.5–−32.0 eV, and −7.48–9.42 eV, for the 5s, 4p, and 4d orbitals of niobium and the nearest iron atom 4s, 4p, and 3d orbitals, there are local peaks for the electronic density of states distribution. Overlap of the orbital distribution energy of each atomic orbit is the main sign that atoms participate in bonding. Local peaks in the range of −7.48 to 9.42 eV are the most significant, indicating that the 3d orbital of iron mainly interacts with the 4d orbital of niobium.

**Figure 7:**
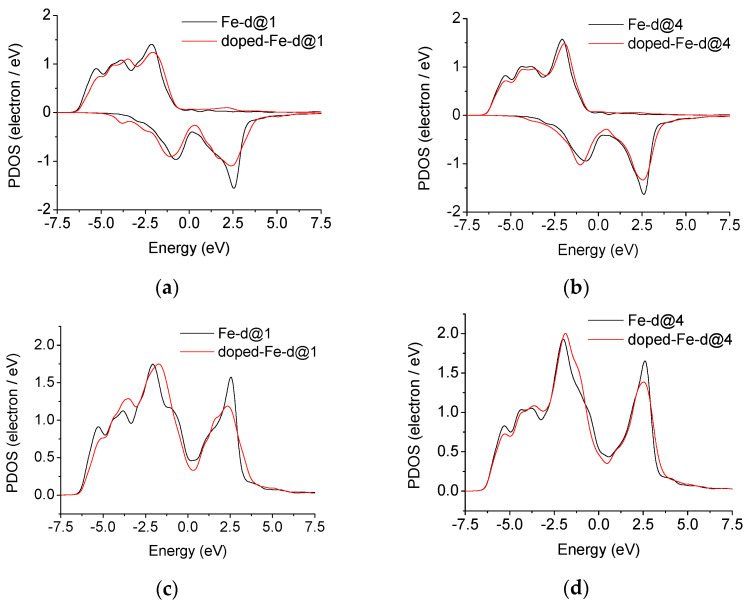
Nb-undoped and Nb-doped electronic density of states of bcc Fe for Σ3[110] (112) grain boundaries; contributions from spin-up and spin-down eigenstates at (**a**)position 1, (**b**) position 4; contributions from both spin-up and spin-down eigenstates summed at (**c**) position 1, (**d**) position 4.

The distribution of the 3d valence electron density of state for iron at the position closest to niobium before and after the addition of niobium is shown in Figure 7a,c and Figure 7b,d, corresponding to position 1 in Figure 1f and position 4 in Figure 1d.

The 3d electronic density of iron contributions from spin-up and spin-down eigenstates are asymmetric, shown as Figure 7a,b, because the calculations for ferromagnetic bcc-Fe were performed including spin polarization. Figure 7c,d show the 3d electronic density of iron contributions from both spin-up and spin-down eigenstates summed. The 3d electronic density of state of iron decreases near the Fermi surface, the peak height of the 3d electronic density of state distribution above the Fermi surface decreases, and the proportion of the peak area below the Fermi surface increases in the bonding zone. The 3d electronic density of state is distributed at lower energy, so that the energy of the system is lower, the bond is stronger, and the structure is more stable [15,16,36].

Integrating the part below the Fermi surface in Figure 7c, the charges in the occupancy of the 3d state for iron before and after the addition of niobium are 6.56 and 6.67, respectively, an increase of 1.68%. This value and increase are similar to the first-principle calculation value of the charge distribution in Table 5 and the result of the charges in the occupancy of 3d state analyzed according to the electron energy loss spectrum test in Table 2.

From the results of these tests, niobium changes the valence electron structure of iron at grain boundaries, provides more electrons for grain boundary bonding, and enhances grain boundary bonding. The strengthening of grain boundaries by niobium can finally be traced to the changes in the valence electron structure of iron at the grain boundaries: due to the interaction between the 3d orbital of iron and the 4d orbital of niobium, the distribution of the 3d valence electron density of state for iron moves to a lower energy, the charges in the occupancy of the 3d state for iron increases, and the electrons are transferred from niobium to the nearest neighbor iron atom; the interaction between adjacent iron atoms across the grain boundary and on both sides of the grain boundary is strengthened, and the connection between adjacent iron atoms is closer. There is an inherent connection between the distribution of the 3d valence electron density of state for the grain boundary iron atoms and the performance of the material. Therefore, it may be inferred that any element, not limited to niobium, can strengthen the grain boundaries of ferrite and can be used to improve the strength and toughness of steel materials, as long as it can promote the distribution of the 3d valence electron density of state for iron at the grain boundary to the lower energy region. This is very helpful in optimization of material chemical composition design.

## 4. Conclusions

In this paper, the effect of niobium on ferrite grain boundary strengthening is studied from the perspective of the valence electron structure. The segregation of niobium at the grain boundary was observed in the ferrite of X70 pipeline steel through a three-dimensional atom probe test. The maximum atomic percentage content of niobium in the grain boundary reached 0.294 and 0.466 at.%, which is 7.5–11.9 times the niobium content of 0.039 at.% in the matrix. The results of the electron energy loss spectrum test show that the charges in the occupancy of 3d state for iron at the grain boundary are higher than that inside the grain, indicating that the grain boundary bonding was enhanced. From the first-principles calculations, it can be seen that the enhancement of grain boundary bonds is inherently related to the change of the 3d valence electron state density distribution of iron at the grain boundary. Due to the interaction between the 3d orbital of iron and the 4d orbital of niobium, the distribution of the electron density of state moves to a lower energy region, and the charges in the occupancy of 3d state for iron increase. These provide more electrons for grain boundary bonding, thereby enhancing grain boundary bonding, which is beneficial in improvng the strength and toughness of the material. In order to use niobium more effectively to improve the properties of steel, the manufacturing process could be improved to promote the grain boundary segregation of niobium. In addition, the element or combination of elements could be selected from more types of microalloying elements to effectively promote the distribution of the 3d valence electron density of state for iron at the grain boundary to the low energy region, so as to provide more microalloying options for the production of higher strength and toughness steel materials.

## Figures and Tables

**Table 1:** Chemical comparison of X70 steel and the three-dimensional atom probe (3DAP) result in a grain.

Element	C	Si	Mn	P	Nb
Composition (wt.%)	0.065	0.20	1.55	0.016	0.065
Composition (at.%)	0.300	0.39	1.57	0.029	0.039
APT result inside grain (at.%)	0.153	0.56	1.49	0.034	0.032
Maximum concentration of sample 1 (at.%)	2.110	1.27	2.50	0.220	0.466
Maximum concentration of sample 2 (at.%)	2.160	1.29	1.99	0.188	0.294

**Table 2:** Charges in the occupancy of the 3d state for Fe calculated according to EELS.

Position	Grain Boundary	Adjacent Grain1	Adjacent Grain2
1	7.25	7.22	7.24
2	7.57	7.44	7.18
3	7.29	7.24	7.05

**Table 3:** Calculated segregation energies of the system with Nb doped in the grain boundary of bcc Fe (eV).

$E_{GB}^{nFe+Nb}$	$E_{relaxed\;FS}^{\frac{n}{2}Fe+Nb}$	$E_{bulk}^{nFe+Nb}$	$E_{GB}^{nFe}$	$E_{relaxed\;FS}^{\frac{n}{2}Fe}$	$E_{form}^{GB}$	$E_{form}^{clean}$	$E_{form}^{bulk}$	$\Delta E_{SE}^{GB}$	$\Delta E_{SE}^{bulk}$	$\Delta E_{SE}^{FS}$
−377.46	−178.38	−376.98	−370.30	−178.34	−20.74	−13.63	−20.26	−7.12	−6.64	−0.04

**Table 4:** Change of bond length between adjacent iron atoms of niobium before and after doping (Å).

Bond	0–1	4–5	7–6	8–9	11–10	4–8	5–9	6–10	7–11
undoped	2.482	2.420	2.420	2.420	2.420	2.482	2.482	2.482	2.482
Nb-doped	2.461	2.396	2.388	2.387	2.385	2.453	2.462	2.462	2.468

**Table 5:** Mulliken electronic populations of Nb and its neighbor Fe atoms.

Atom	Orbital	Fe (Nb)	Fe1	Fe4	Fe8
Undoped	4s	0.62	0.62	0.62	0.62
	4p	0.83	0.83	0.79	0.79
	3d	6.57	6.57	6.59	6.59
	total	8.03	8.03	8.0	8.0
	Charge/e	−0.03	−0.03	0	0
Nb-doped	4s (5s)	2.8	0.71	0.69	0.64
	4p (4p)	4.94	0.77	0.73	0.73
	3d (4d)	4.01	6.68	6.66	6.62
	Total	11.75	8.16	8.08	7.99
	Charge/e	1.25	−0.16	−0.08	0.01

## Data Availability

Data sharing is not applicable to this article.
